# Effectiveness of prophylactic levosimendan in high-risk valve surgery patients

**DOI:** 10.5830/CVJA-2013-047

**Published:** 2013-10

**Authors:** Ozgur Ersoy, Emre Boysan, Ertekin Utku Unal, Kerem Yay, Umit Yener, Ferit Cicekcioglu, Fehmi Katircioglu

**Affiliations:** Department of Cardiovascular Surgery, Turkey Yuksek Ihtisas Hospital, Ankara, Turkey; Department of Cardiovascular Surgery, Turkey Yuksek Ihtisas Hospital, Ankara, Turkey; Department of Cardiovascular Surgery, Turkey Yuksek Ihtisas Hospital, Ankara, Turkey; Department of Cardiovascular Surgery, Turkey Yuksek Ihtisas Hospital, Ankara, Turkey; Department of Cardiovascular Surgery, Turkey Yuksek Ihtisas Hospital, Ankara, Turkey; Department of Cardiovascular Surgery, Turkey Yuksek Ihtisas Hospital, Ankara, Turkey; Department of Cardiovascular Surgery, Ankara Hospital, Ankara, Turkey

**Keywords:** cardiac valve, cardiac surgery, pulmonary hypertension, levosimendan, left ventricular dysfunction

## Abstract

**Background:**

Levosimendan has anti-ischaemic effects, improves myocardial contractility and increases systemic, pulmonary and coronary vasodilatation. These properties suggest potential advantages in high-risk cardiac valve surgery patients where cardioprotection would be valuable. The present study investigated the peri-operative haemodynamic effects of prophylactic levosimendan infusion in cardiac valve surgery patients with low ejection fraction and/or severe pulmonary arterial hypertension.

**Methods:**

Between May 2006 and July 2007, 20 consecutive patients with severe pulmonary arterial hypertension (systolic pulmonary artery pressure ≥ 60 mmHg) and/or low ejection fraction (< 50%) who underwent valve surgery in our clinic were included in the study and randomised into two groups. Levosimendan was administered to 10 patients in group I and not to the 10 patients in the control group. Cardiac output (CO), cardiac index (CI), systemic vascular resistance (SVR), pulmonary vascular resistance (PVR) and mean pulmonary artery pressure (MPAP) were recorded for each patient preoperatively and for 24 hours following the operation.

**Results:**

CO and CI values were higher in the levosimendan group during the study period (*p* < 0.05). MPAP and PVR values were significantly lower in the levosimendan group for the 24-hour period (*p* < 0.05) and SVR values were significantly lower after 24 hours in both groups. When clinical results were considered, no difference in favour of levosimendan was detected regarding the mortality and morbidity rates between the groups.

**Conclusion:**

Levosimendan improved the haemodynamics in cardiac valve surgery patients with low ejection fraction and/or severe pulmonary arterial hypertension, and facilitated weaning from cardiopulmonary bypass in such high-risk patients when started as a prophylactic agent.

## Abstract

Pulmonary arterial hypertension and low ejection fraction were among the key factors determining prognosis during the postoperative period in patients with cardiac valve disease who underwent cardiac surgery.^1^,^2^ Left ventricular dysfunction, which makes weaning from cardiopulmonary bypass (CPB) difficult and increases morbidity and mortality rates, may develop in the patient group that has either or both pulmonary arterial hypertension and low ejection fraction.

Levosimendan is a recently introduced calcium sensitiser. It enhances myocardial contractility by sensitisation of troponin C to calcium, and provides systemic, pulmonary and coronary arterial and venous vasodilatation due to activation of the ATP-sensitive potassium channels in smooth muscle fibres.^3^ It has positive inotropic and anti-stunning effects.^4^,^5^ It has been reported that levosimendan facilitated weaning from CPB in high-risk patient groups by reducing pulmonary arterial pressure and increasing both right and left ventricular contractility, which means improved ejection fraction and cardiac output.^6^,^7^

In the present study, we documented haemodynamic changes caused by levosimendan infusion, instituted just after the induction of anaesthesia, as a measure in cardiac valve surgery patients with low ejection fraction and/or pulmonary arterial hypertension.

## Methods

Between May 2006 and July 2007, 20 consecutive patients with severe pulmonary arterial hypertension (systolic pulmonary artery pressure ≥ 60 mmHg) and low ejection fraction (< 50%) who underwent valve surgery in our clinic, were included in the study and randomised to two groups (levosimendan and control groups). The conventional definition of pulmonary arterial hypertension includes mean pulmonary arterial pressure of > 25 mmHg at rest as assessed by right heart catheterisation. Our study group of patients was selected as having severe pulmonary hypertension, which was defined in our clinical practice as systolic pulmonary artery pressure ≥ 60 mmHg.

This study complied with the Declaration of Helsinki and ethical approval was granted by the local institutional review board. Informed consent was obtained from all patients.

The anaesthetic and surgical management of all patients was the same in both groups. Induction and maintenance of general anaesthesia with endotracheal intubation was standardised in all the patients (sufentanil, midazolam, pancuronium or atracurium, and sevoflurane in oxygen with air). Invasive haemodynamic monitoring, including thermodilution catheterisation, was established, allowing for haemodynamic measurements at different time points.

Myocardial protection was obtained by cold blood cardioplegic solution. Patients were cooled to 32°C applying alpha-stat acid–base management. Perfusion pressure was maintained in the range of 40 to 70 mmHg.

Cardiac output (CO), cardiac index (CI), systemic vascular resistance (SVR), pulmonary vascular resistance (PVR) and mean pulmonary artery pressure (MPAP) were recorded for each patient. Levosimendan (Simdax; Orion Corporation, Finland) was administered to 10 patients following anaesthetic induction, with a loading dose of 12 μg/kg administered in 10 minutes, followed by a 24-hour infusion at a rate of 0.1 μl/kg/min (group I). Ten patients to whom levosimendan was not administered were regarded as the control group (group II).

Measurements were performed using a 7F Multiflex thermodilution catheter (Abbot Laboratories, Hospital Products Division, USA). Cold normal saline was administered through the proximal end of the thermodilution catheter and sampling was performed from the distal end of the catheter. Five measurements were carried out for each parameter, minimum and maximum values were excluded, and averages of the remaining values were obtained. On the other hand, PVR and SVR values were calculated and recorded by the computer system.

Following insertion of the thermodilution catheter after general anaesthesia, initial values were recorded for all the patients and these were regarded as baseline values (CO_1_, CI_1_, SVR_1_, PVR_1_, and MPAP_1_). The rest of the measurements in the levosimendan group were acquired following the administration of the loading dose (CO_2_, CI_2_, SVR_2_, PVR_2_, MPAP_2_), at the sixth hour of the levosimendan infusion (CO_3_, CI_3_, SVR_3_, PVR_3_, MPAP_3_), at the 12th hour of levosimendan infusion (CO_4_, CI_4_, SVR_4_, PVR_4_, MPAP_4_), and at 24th hour of the levosimendan infusion (CO_5_, CI_5_, SVR_5_, PVR_5_, MPAP_5_). Measurements for the control group were performed at equivalent periods.

## Statistical analysis

Normally distributed continuous variables were expressed as mean values ± standard deviation (SD). Categorical variables were expressed as numbers and percentages. Demographic characteristics, peri-operative variables and calculated values were compared using independent samples *t*-test for continuous variables and the chi-square test or Fisher’s exact test for categorical variables. Within-group differences were evaluated with the paired-samples *t*-test. A *p*-value < 0.05 was considered statistically significant. All statistical analyses were performed using the SPSS statistical software (SPSS for Windows 12.0, Inc., Chicago, IL, USA).

## Results

Demographic data of patients in the levosimendan and control groups are shown in Table 1. There was no difference between the two groups apart from body surface area values. In addition, there was no difference between EuroSCORE values of groups I and II (*p* = 0.418).

**Table 1:** Comparison Of Demographic Data Between Groups

*Characteristics*	*Group I (levosimendan) (n = 10)*	*Group II (control) (n = 10)*	p*-value*
Age (years)	49.6 ± 10.7	45.7 ± 7.9	0.125
Male/female	5/5	3/7	0.361
BSA (m^2^)	1.60 ± 0.22	1.67 ± 0.17	0.006
Functional capacity (NYHA)	3.2 ± 0.6	3.4 ± 0.5	1.000
Pre-operative EF (%)	46.8 ± 10.9	49.0 ± 12.0	0.182
COPD (+/–)	3/7	2/7	0.695
Pre-operative sPAP (mmHg)	71.2 ± 23.6	72.8 ± 15.8	0.151

BSA: body surface area, COPD: chronic obstructive pulmonary disease, EF: ejection fraction, NYHA: New York Heart Association, sPAP: systolic pulmonary arterial pressure.

In group I, there were three patients with mitral regurgitation, four with mitral stenosis and three with combined aortic stenosis and mitral stenosis. On the other hand, in group II, there were six patients with mitral stenosis, two with combined aortic stenosis and mitral stenosis and two with prosthetic valve dysfunction. Surgical procedures performed on both groups are summarised in Table 2.

**Table 2:** Surgical Procedures Performed

*Surgery type*	*Group I (levosimendan)*	*Group II (control)*
MVR (redo)	2	2
MVR	2	4
AVR + MVR	3	2
Mitral repair	1	0
Mitral repair + CABG	2	0
AVR + MVR (redo AVR)	0	1
AVR + MVR (redo MVR)	0	1

MVR: mitral valve replacement, AVR: aortic valve replacement, CABG: coronary artery bypass graft.

Duration of cross-clamp, CPB and surgery, dosage of inotropic drugs, and the length of intensive care unit and hospital stay of both groups are documented in Table 3. There was no significant difference between cross-clamp, CPB and operation times. No difference was detected for length of intensive care unit stay, whereas it was found that the length of hospital stay for the study group was significantly longer (group I: 7.8 ± 2.4 days vs group II: 5.8 ± 1.5 days; *p* = 0.014). No marked adverse reaction to the drug was observed in group I.

**Table 3:** Intra- And Postoperative Data

*Features*	*Group I*	*Group II*	p*-value*
XCL period (min)	88.7 ± 56.4	69.2 ± 26.8	0.779
CPB period (min)	115.4 ± 62.8	89.4 ± 33.2	0.884
Operation time (min)	219.5 ± 83.2	155.0 ± 49.4	0.424
Need for inotropic drug	5	2	0.160
Need for IABP	0	0	–
Mortality	0	0	–
Postoperative exploration	0	0	–
Low cardiac output	0	0	–
Acute renal failure	0	0	–
Length of stay in ICU (days)	2.7 ± 2.1	1.4 ± 1.3	0.893
Length of stay at hospital (days)	7.8 ± 2.4	5.8 ± 1.5	0.012

XCL: cross-clamp, CPB: cardiopulmonary bypass; IABP: intra-aortic balloon counterpulsation, ICU: intensive care unit.

**Fig. 1.**
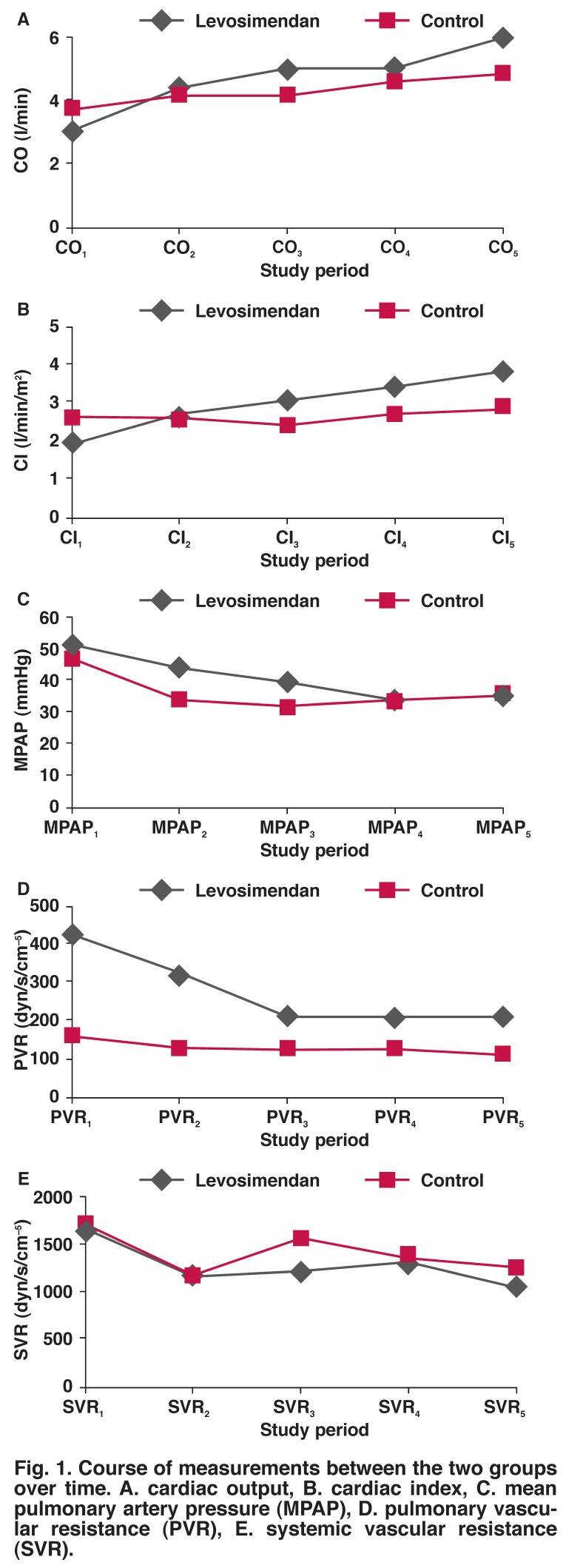
Course of measurements between the two groups over time. A. cardiac output, B. cardiac index, C. mean pulmonary artery pressure (MPAP ), D. pulmonary vascular resistance (PVR), E. systemic vascular resistance (SVR).

A statistically significant difference in favour of the levosimendan group was recorded regarding the statistical values of cardiac outputs and cardiac indexes between the two groups. For baseline values, CO1 values of group I were significantly lower than those of group II (group I: 3.02 ± 0.36 l/min vs group II: 3.71 ± 0.92 l/min; *p* = 0.001) (Fig. 1A). Considering all measurements, CO_3_ and CO_5_ for group I were higher than those of the control group, whereas there was no difference for the other measurements [respectively for groups I and II; CO_2_: 4.39 ± 1.56 vs 4.18 ± 0.72 l/min (*p* = 0.804); CO_3_: 5.01 ± 0.57 vs 4.11 ± 1.00 l/min (*p* = 0.024); CO_4_: 5.03 ± 1.01 vs 4.62 ± 0.61 l/min (*p* = 0.191); CO_5_: 5.94 ± 1.14 vs 4.87 ± 0.34 l/min (*p* = 0.049)] (Fig. 1A).

When within-group CO increase was evaluated, CO in the levosimendan group showed a significant increase with time compared to baseline values (CO_1_: 3.02 ± 0.36 l/min vs CO_5_: 5.94 ± 1.14 l/min; *p* = 0.018). On the other hand, increase in the control group over time was not found to be significant (CO_1_: 3.71 ± 0.92 l/min vs CO_5_: 4.87 ± 0.34 l/min; *p* = 0.506).

Statistically significant differences in favour of group I were recorded regarding the values of CI between the two groups. CI in group I increased significantly compared to the control group [respectively for groups I and II; CI_2_: 2.68 ± 0.83 vs 2.54 ± 0.47 l/min/m^2^ (*p* = 0.273); CI_3_: 3.13 ± 0.37 vs 2.40 ± 0.54 l/min/^2^ (*p* = 0.229); CI_4_: 3.43 ± 0.66 vs 2.74 ± 0.31 l/min/^2^ (*p* = 0.006); CI_5_: 3.84 ± 0.81 vs 2.94 ± 0.29 l/min/^2^ (*p* = 0.001)] (Fig. 1B).

When the within-group CI increase was evaluated, CI in group 1 showed a significant increase over time compared to baseline values (CI_1_: 1.89 ± 0.30 l/min/m^2^ vs CI_5_: 3.84 ± 0.81 l/min/^2^; *p* = 0.014). Although the increase in group II over time was found to be significant (CI1: 2.60 ± 1.26 vs 2.94 ± 029 l/min/^2^; *p* = 0.048) this increase was more apparent in group I.

Basal pulmonary arterial pressures were compared (PAP_1_) between groups. PAP_1_ in group I was higher compared to that in group II (respectively for groups I and II; PAP1: 51.25 ± 26.95 vs 47.00 ± 9.00 mmHg; *p* = 0.001). PAP_1_ was decreased significantly in group I over time (PAP_1_: 51.25 ± 26.95 mmHg vs PAP_5_: 36.00 ± 12.56 mmHg; *p* = 0.032). This decrease was not significant in the control group (PAP_1_: 47.00 ± 9.00 mmHg vs PAP_5_: 35.85 ± 8.29 mmHg; *p* = 0.595) (Fig. 1C).

When basal pulmonary vascular resistance values (PVR_1_) were compared, values in group I were higher compared to those in group II (respectively for groups I and II; PVR_1_:432.4 ± 340.4 vs 164.2 ± 79.5 dyne/s/cm^5^; *p* = 0.027). The decrease in PVR over time was marked in group I (PVR_1_: 432.4 ± 340.4 dyne/s/cm^5^ vs PVR_1_: 218.7 ± 163.2 dyne/s/cm^5^; *p* = 0.009). This decrease was not significantly different with time in the control group (PVR_1_: 164.2 ± 79.5 dyne/s/cm^5^ vs PVR_5_: 116.1 ± 49.6 dyne/s/cm^5^; *p* = 0.445) (Fig. 1D).

Baseline systemic vascular resistance values (SVR_1_) were compared between the groups. In group II, SVR_1_ was higher than that in group I (respectively for groups I and II; SVR_1_: 1681.2 ± 422.6 vs 1740.0 ± 698.5 dyne/s/cm^5^, *p* = 0.032). Decrease in SVR with time was significant in group I (SVR_1_: 1681.2 ± 422.6 dyne/s/cm^5^ vs SVR_5_: 1039.2 ± 354.2 dyne/s/cm^5^; *p* = 0.015). In the control group, SVR_1_ also showed a significant decrease (SVR_1_: 1740.0 ± 698.5 dyne/s/cm^5^ vs SVR5: 1272.2 ± 375.5 dyne/s/cm^5^; *p* = 0.036) (Fig. 1E).

## Discussion

Nowadays many patients indicated for cardiac surgery are at high peri-operative risk for increased risk of morbidity and mortality. Pulmonary arterial hypertension and low ejection fraction are among the key factors determining prognosis in the postoperative period in patients with valve diseases.^1^,^2^ Individually or combined, the presence of these risk factors may make the weaning from CPB difficult and may cause severe left and right ventricular failure after the CPB procedure.

Treatment methods for patients who cannot be weaned from CPB or develop low cardiac output after CPB include use of inotropic agents, vasodilators, intra-aortic balloon pump, insertion of a balloon pump into the pulmonary artery, implementation of right ventricular assist devices and extracorporeal membrane oxygenisation. A newly developed agent, levosimendan, is now available. It exhibits positive inotropic activity by increasing the ionised calcium sensitivity of cardiac troponin C and facilitating calcium binding to the myofilaments. Additionally, it exhibits vasodilator effects on the decrease in intracellular calcium level by allowing the ATP-sensitive potassium channels to be opened.^3^ Levosimendan differs from other positive inotropic drugs with features such as increasing contractility without increasing myocardial oxygen consumption, improving coronary perfusion with its vasodilator activity, reducing preload and afterload by vasodilatation in the pulmonary, renal, splanchnic, cerebral and systemic arteries as well as in the saphenous, portal and systemic veins.^8^,^9^

There are many reported studies of levosimendan being used in cardiac surgery. In many of these studies, levosimendan was started after cardiac surgery or during CPB weaning.^6^ In only a few studies, levosimendan was started before CPB.

Tritapepe *et al.* reported that a short infusion of levosimendan before coronary artery bypass grafting (CABG) protected the myocardium and improved postoperative haemodynamics. Levosimendan-treated patients had lower postoperative troponin I concentrations and a higher cardiac index, suggestive of a preconditioning effect.^10^

Leppikangas *et al.* administered levosimendan to patients who underwent combined aortic valve and coronary bypass surgery for 24 hours before surgery. They found that both CI and stroke volume were higher in the levosimendan group and concluded that in patients undergoing risky cardiac surgery, levosimendan improved haemodynamics compared with placebo.^11^

Brezina *et al.* showed that levosimendan infusion after the induction of general anaesthesia in high-risk cardiac surgery patients resulted in better outcomes for the length of hospital stay and 30-day mortality rate, compared with patients receiving dobutamine and milrinone.^12^ In another study by Tritapepe *et al.*, intravenous bolus administration of levosimendan over a 10-minute period before initiation of bypass resulted in less myocardial injury, a reduction in tracheal intubation time, less requirement for inotropic support and a shorter length of intensive care unit stay, compared with placebo.^13^

In our work, a dose titration study showed that even at a minimal dosage of levosimendan, an increase of approximately 12 ml in stroke volume and an increase of 0.7 l/min/m^2^ in cardiac index were found. When compared with placebo, levosimendan caused a significantly higher positive haemodynamic response at the sixth hour (17% with placebo, 80% with levosimendan). Symptomatic improvement in patients has been found to be parallel to haemodynamic improvement. Folloth *et al*. demonstrated that positive haemodynamic responses continued for 24 hours following discontinuation of the infusion.^14^

In our study, cardiac output and cardiac index values in the levosimendan group were significantly lower compared to the control group. Cardiac output and cardiac index in the levosimendan group also showed remarkable increases compared to the control group. In patients receiving levosimendan, at the end of the 24th hour, an increase of approximately 2.9 l/min in cardiac output and an increase of approximately 2 l/min/m^2^ in cardiac index were found. In the control group, at the end of the 24th hour, this increase remained limited to approximately 0.7 l/min in cardiac output and 0.3 l/min/m^2^ in cardiac index. Correspondingly, Tachibana *et al.* showed that levosimendan improved left ventricular systolic and diastolic performance at rest and during exercise after heart failure.^15^

In our study, we also examined pulmonary arterial pressure, pulmonary vascular resistance and systemic vascular resistance in order to determine the vasodilatatory effect of levosimendan. When considered individually, initial values of mean pulmonary arterial pressure were found to be significantly higher in the levosimendan group. At the end of the 24th hour, a marked decrease in the pulmonary arterial pressure was observed in the levosimendan group, but the decrease in the control group was not significant.

A marked decrease in pulmonary vascular resistance values was also recorded in favour of levosimendan at the end of the 24th hour. These results were consistent with those of Lilleberg *et al.*, who found that levosimendan decreased pulmonary vascular resistance early after CABG.^16^ Systemic vascular resistance values showed marked decreases in both the levosimendan and control groups.

Pre-operative cardiac output values in the levosimendan group were less than those of the control group, whereas pulmonary vascular resistance and pulmonary pressure values measured by thermodilution catheter were significantly higher compared to the control group. Despite these values, this patient group was easily weaned from CPB and the postoperative period went smoothly. This patient group was discharged with a full recovery.

More significant results were obtained from the levosimendan group compared to the control group regarding increases in postoperative CO and CI, and decreases in PVR and PAP values. Based on the published literature mentioned above, we expected that greater preservation of cardiac function after CPB would result in a better recovery. Our results were consistent with these findings. This indicates that levosimendan was beneficial. However, when clinical results were considered, no difference in favour of levosimendan was determined regarding the mortality and morbidity rates between the groups.

The most common adverse reactions with levosimendan include headache, dizziness, hypotension, ventricular tachycardia, atrial fibrillation, tachycardia, ventricular extrasystoles, cardiac failure, myocardial ischaemia, extrasystoles, nausea, vomiting, constipation, diarrhoea, insomnia, decreased haemoglobin and hypokalaemia. There was no significant adverse effect from the drug in our study group.

When the groups were compared with regard to risk scoring (EuroSCORE), they were found to be similar. On the other hand, the pre-operative CO and CI values were lower and the PVR and PAP values were higher in the levosimendan group, but it will be noted that this was a more high-risk group. Despite this, this group could easily be weaned from CPB and discharged with a full recovery.

In our study, patients in the levosimendan group were discharged later than the control group, contrary to results in the literature. We surmise this was because of their more risky profiles despite the fact that their EuroSCOREs were similar to those of the control group. Rates of mortality and morbidity were found to be similar in both groups, possibly due to the small number of patients in the study.

## Conclusion

Our study shows that pre-operative use of levosimendan in cardiac valve surgery patients with low ejection fractions and/or pulmonary arterial hypertension resulted in improved haemodynamic parameters, which may have provided better and faster recovery after CPB. In larger studies with more patients, the positive effects of levosimendan on clinical outcomes may be seen more clearly.
